# Promising clinical and immunological efficacy of *Bacillus clausii* spore probiotics for supportive treatment of persistent diarrhea in children

**DOI:** 10.1038/s41598-024-56627-9

**Published:** 2024-03-18

**Authors:** Ha Thuy Dang, Dien Minh Tran, Thuy Thi Bich Phung, Anh Thi Phuong Bui, Yen Hai Vu, Minh Thi Luong, Hang Minh Nguyen, Huong Thi Trinh, Tham Thi Nguyen, Anh Hoa Nguyen, Anh Thi Van Nguyen

**Affiliations:** 1 Department of Gastroenterology, Vietnam National Children’s Hospital, No. 18/879 La Thanh, Dong Da, Hanoi, Vietnam; 2 Department of Surgical Intensive Care Unit, Vietnam National Children’s Hospital, No. 18/879 La Thanh, Dong Da, Hanoi, Vietnam; 3 Department of Molecular Biology for Infectious Diseases, Vietnam National Children’s Hospital, No. 18/879 La Thanh, Dong Da, Hanoi, Vietnam; 4 Spobiotic Research Center, ANABIO R&D Ltd. Company, No. 22, Lot 7,8 Van Khe Urban, La Khe, Ha Dong, Hanoi, Vietnam; 5 LiveSpo Pharma Ltd. Company, N03T5, Ngoai Giao Doan Urban, Bac Tu Liem, Hanoi, Vietnam

**Keywords:** Persistent diarrhea, Probiotic, *Bacillus clausii*, Spore, Cytokine, Randomized controlled trials, Diarrhoea, Interleukins, Paediatric research, Applied microbiology

## Abstract

Persistent diarrhea is a severe gastroenteric disease with relatively high risk of pediatric mortality in developing countries. We conducted a randomized, double-blind, controlled clinical trial to evaluate the efficacy of liquid-form *Bacillus clausii* spore probiotics (LiveSpo CLAUSY; 2 billion CFU/5 mL ampoule) at high dosages of 4–6 ampoules a day in supporting treatment of children with persistent diarrhea. Our findings showed that *B. clausii* spores significantly improved treatment outcomes, resulting in a 2-day shorter recovery period (*p* < 0.05) and a 1.5–1.6 folds greater efficacy in reducing diarrhea symptoms, such as high frequency of bowel movement of ≥ 3 stools a day, presence of fecal mucus, and diapered infant stool scale types 4-5B. LiveSpo CLAUSY supportive treatment achieved 3 days (*p* < 0.0001) faster recovery from diarrhea disease, with 1.6-fold improved treatment efficacy. At day 5 of treatment, a significant decrease in blood levels of pro-inflammatory cytokines TNF-α, IL-17, and IL-23 by 3.24% (*p* = 0.0409), 29.76% (*p* = 0.0001), and 10.87% (*p* = 0.0036), respectively, was observed in the Clausy group. Simultaneously, there was a significant 37.97% decrease (*p* = 0.0326) in the excreted IgA in stool at day 5 in the Clausy group. Overall, the clinical study demonstrates the efficacy of *B. clausii* spores (LiveSpo CLAUSY) as an effective symptomatic treatment and immunomodulatory agent for persistent diarrhea in children.

Trial registration: NCT05812820.

## Introduction

Diarrhea is a common gastrointestinal disorders characterized by bowel movement of three or more times a day and watery or liquid stool, which is particularly prevalent in children^1^. It is also one of the leading causes of morbidity and mortality in children under five years of age, with 1.7 billion episodes and 578,000 deaths recorded annually worldwide^2,3^. During waves of different COVID-19 variant infections, the rate of diarrhea among children tends to increase. Pediatric COVID-19 patients often present with diarrhea, and the symptom is linked to heightened systemic inflammation^4^. World Health Organization (WHO) definition of persistent diarrhea includes cases of diarrhea, lasting between 2 and 4 weeks, and excludes chronic diarrhea caused by other conditions, such as celiac disease, food‐related enteropathies, and congenital enteropathies. Persistent diarrhea accounts for approximately 3% to 20% of all diarrhea episodes in children under the age of five and can result in severe dehydration, malnutrition, and hospitalization if left untreated^5,6^. The consequences of persistent diarrhea extend beyond the individual child and affect the nutritional status, treatment costs, and public health of communities worldwide. While the mortality rate associated with acute diarrhea is decreasing, the rate of deaths caused by persistent diarrhea has increased. Despite constituting a relatively small proportion of cases, it significantly contributes to diarrhea morbidity, accounting for up to 50% of all deaths related to diarrhea^7^. Due to the difference in the major causes of persistent diarrhea between developed and developing countries, it is critical to have different approaches to diagnosis and management. In developing countries, it is usually associated with repeated enteric infections that result in malnutrition, particularly in children. Additionally, weakened immune systems in children resulting from persistent diarrhea can lead to other illnesses, such as respiratory infections. In contrast, in developed countries, persistent diarrhea is more likely a consequence of underlying diseases, such as inflammatory bowel disease. Although children in these cases are less likely to be exposed to multiple enteric infections, persistent diarrhea can still lead to malnutrition and other health problems if left untreated^8,9^. Oral rehydration solution, zinc, and antibiotics are the most common treatments for children hospitalized with diarrhea, largely following WHO guidelines^10^.

In recent years, significant progress has been made in understanding the relationship between diarrhea, gut microflora imbalance, and intestinal immunity. Inflammation is protective immune responses mobilizing both the innate and adaptive arms to eliminate invading pathogens and other harmful stimuli. Diarrhea, although not every case of it displays acute inflammation^11^, is a hallmark of intestinal inflammation^12^. It is characterized by increased pro-inflammatory cytokines such as IL-1β, IL-6, IFN-γ, TNF-α and decreased anti-inflammatory cytokines (IL-4, IL-10)^13,14^. In the adaptive arm of intestinal immune system, T-lymphocytes in the Th17 response^15–17^ and IgA-producing B-lymphocytes^18,19^ are key cells in regulating gut homeostasis and its interacting with microbiota in the normal state and during diarrhea.

In the past two decades, a number of randomized controlled trials has demonstrated clinical benefit and safety of a wide range of probiotics in prevention and control of gastrointestinal diseases^20–24^. However, the effects of probiotics in children with persistent diarrhea are much less studied compared to acute diarrhea. A meta-analysis by Aponte et al. (2013) was conducted to review four trials investigating the efficacy of *Lactobacillus* sp. and *Saccharomyces boulardii* probiotics in children with persistent diarrhea (*n* = 464). The analysis revealed a statistically significant 4-day reduction in the duration of persistent diarrhea with probiotic intervention (*n* = 324, 2 trials). Additionally, stool frequency was reported to be reduced in two of the trials. Notably, one trial showed a significant decrease in hospital stay^25^.

Compared to the aforementioned probiotic species, *Bacillus* probiotics have an advantage due to their ability to form endospores that are resistant to environmental stressors such as heat, acidic gastric pH, and bile. These features enable *Bacillus* probiotics to survive better in the harsh conditions of the digestive tract and reach the intestines in an intact form, where they can provide health benefits. Among safe *Bacillus* species, *B. clausii* has been recommended for managing disruptions in the intestinal bacterial flora in both children and adults. *B. clausii* has been shown to decrease rotavirus/adenovirus excretion and reduce stool frequency in children with acute diarrhea^26,27^. Results from six randomized controlled trials with 1298 patients showed a substantial decrease in both the duration of diarrhea (mean difference = −9.12 h) and hospitalization (mean difference = −0.85 day) in pediatric acute diarrhea treated with *B. clausii*. Additionally, there was a trend towards reduced stool frequency (mean difference = −0.19 bowel movement), indicating that *B. clausii* may serve as an effective and safe therapeutic option for managing acute diarrhea in children^27^. For more than 55 years, *B. clausii* has been available on the market and is distinguished by the presence of four probiotic strains including O/C, SIN, N/R and T^28^. These combined *B. clausii* strains in one probiotic product exhibit high levels of poly-antibiotic resistance, ensuring their effectiveness even when used in conjunction with antibiotic treatment^29^. The *B. clausii* strains at daily dose of two ampoules × two billion CFU/ampoule were effective in reducing the average number of diarrhea episodes during the treatment period. Specifically, the median number of episodes per day decreased from 5 at the start of treatment to 1 on the fifth day of treatment, which was a significant improvement^30^. In another recent randomized clinical trial by Maity and Gupta (2021), *B. clausii* 088AE strain was effective in alleviating acute antibiotic-associated diarrhea (AAD) in both pediatric and adolescent/adult groups. The probiotics have significantly improved diarrhea conditions, reduced AAD severity scores, and was well-tolerated with no reported adverse effects. The results suggest that this probiotic strain is a safe and effective live bio-therapeutic agent for managing AAD and associated symptoms^31^.

Most clinical trials of *B. clausii* have been conducted on children with acute diarrhea using recommended dosages of 2–4 billion CFU daily. In addition, no trials of *B. clausii* have been conducted on children with persistent diarrhea so far, possibly due to the more severe and difficult-to-treat symptoms. To date, no studies have evaluated the efficacy of probiotics on the immune response in children with persistent diarrhea, specifically through the measurement of clinical indicators such as IgA and pro-inflammatory cytokines which are associated with gut immune function^32–34^. In this study, we proposed that a spore probiotic of *B. clausii* ANA39 strain (LiveSpo CLAUSY, LiveSpo Pharma) supplementation at two to threefold higher dose of 8–12 billion CFU daily than conventional dose, can be used as a potential solution for treating patients with persistent diarrhea. We conducted a randomized double-blind controlled clinical study to evaluate the efficacy of this supportive treatment in children aged from 3 to 24 months. In the study, we recorded the frequency and consistency of the participants' stools. The duration of diarrhea was measured by calculating the number of days from the time of admission until the first normal stool was passed according to the diapered infant stool scale, which considers a score of less than 3 as indicative of normal stool. Additionally, we noted the percentage of patients in each group who experienced ongoing diarrhea after 3 days of treatment. We also evaluated the immune responses by measuring changes in IgA in stool samples and several cytokines, including pro-inflammatory IL-6, TNF-α, IL-17, IL-23, and anti-inflammatory IL-10 in blood samples at day 5 compared to day 0 before treatment.

## Result

### Trial design and patient baseline demographic, clinical and subclinical characteristics

**Figure 1:**
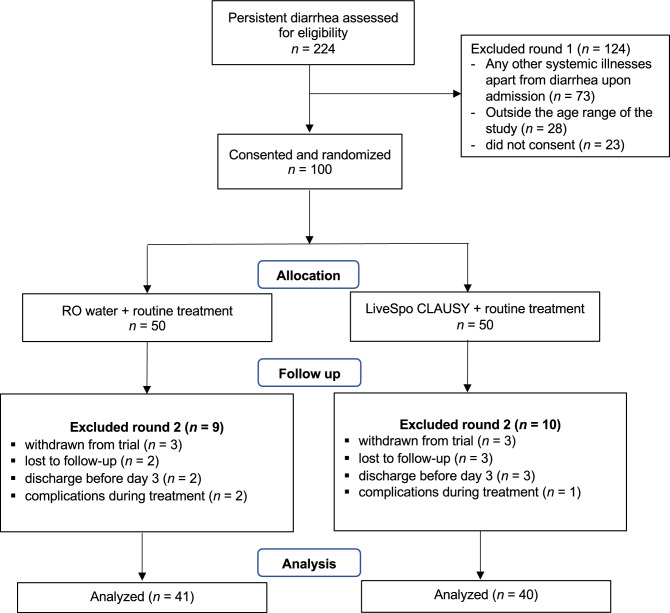
A flowchart illustrating the participant enrolment process in this study. Participants were initially screened for eligibility using the clinical database. Those who met the criteria and provided informed consent were then randomly assigned to either the Control or Clausy group. Data collection and analysis were carried out between day 0 (baseline) and the end of the treatment period. The study recruited participants and conducted clinical and subclinical measurements from July 2022 to July 2023.

From July 2022 to July 2023, a total of 9160 patients with diarrhea visited the hospital for examination. Among them, 4734 patients were admitted for internal treatment. Of these inpatients, 1482 with diarrhea received treatment at the Gastroenterology Department, including 224 with persistent diarrhea who were screened for eligibility to participate in this study. Among them, 100 participants with digestive infection were randomized into two groups consisting of the Control standard of care group (50 patients) and the Clausy testing group (50 patients) (Fig. 1—exclusion round 1). All of these patients lacked a history of frequent probiotic/prebiotic usage (except yogurt) for preventing intestinal diseases, did not use probiotics containing *B. clausii* spores between the onset of diarrhea and prior to hospital admission, and had *B. clausii* negatively detected in stool samples on day 0 by the real-time PCR assay. During the typical 5–10-day follow-up period after treatment, nine participants from the Control group and ten from the Clausy group were excluded, resulting in 41 participants in the Control group and 40 participants in the Clausy group included in the final analysis (Fig. 1—exclusion round 2).

Regarding the baseline demographic characteristics, children with persistent diarrhea participated in this study were between 3 and 24 months old, and there were no significant differences in age or gender distribution between the two groups (*p* = 0.62 and 0.75) (Table 1). Both groups had a high incidence of maternal COVID-19 infection, ranging from 53.66% to 60% (*p* = 0.56). The children themselves had a lower COVID-19 infection rate of about 10%, with no statistically significant difference between the two groups (*p* > 0.9999). The average weight and height of the two groups were similar with no significant difference (*p* = 0.44 and 0.85, respectively). More than 95% of patients in both groups had “Weight for age Z-scores” and “Height for age Z-scores” within the normal range of ≥ −2SD to ≤ 2SD. There was also no statistically significant difference between the two groups regarding the Weight for age Z-scores (all *p* values > 0.9999) and Height for age Z-scores (*p* = 0.49 and > 0.9999). We noted that 14/41 cases (34.15%) in the Control group and 18/40 cases (45.00%) in the Clausy groups had used antibiotics before the onset of diarrhea (*p* = 0.32). The most frequently prescribed antibiotics to treat acute respiratory or intestinal infections prior to the onset of diarrhea were Biseptol, Cefixime, and Augmentin. However, detailed information on the dosage and duration of antibiotic treatment was not available.

**Table 1:** Demographic and clinical characteristics of children with persistent diarrhea before, during, and after treatment.

Characteristic	Control group (N = 41)			Clausy group (N = 40)			*p* value		
	Before treatment	During and after treatment		Before treatment	During and after treatment		Before treatment	During and after treatment	
	Day 0	Day 3	Day 5	Day 0	Day 3	Day 5	Day 0	Day 3	Day 5
Age (months)									
3 ≤ x ≤ 12, *n* (%)	40 (97.56)			38 (95.00)			0.62^a^		
12 < x ≤ 24, *n* (%)	1 (2.44)			2 (5.00)					
Gender									
Male, *n* (%)	24 (58.54)			22 (55.00)			0.75^b^		
Female, *n* (%)	17 (41.46)			18 (45.00)					
Covid-19 infection									
Mother, *n* (%)	22 (53.66)			24 (60.00)			0.56^b^		
Children, *n* (%)	4 (9.76)			3 (7.50)			> 0.9999^a^		
Weight									
Average ± SD	7.24 ± 1.34	7.26 ± 1.34	7.3 ± 1.32	7.03 ± 1.22	7.06 ± 1.21	7.08 ± 1.21	0.44^c^	0.46^c^	0.42^c^
Weight for age Z-score									
< −2SD, *n* (%)	1 (2.44)			0 (0.00)			> 0.9999^a^		
≥ −2SD and ≤ 2SD, *n* (%)	40 (97.56)			40 (100)			> 0.9999^a^		
> 2SD, *n* (%)	0 (0.00)			0 (0.00)					
Height									
Average ± SD	64.63 ± 4.82			64.83 ± 4.09			0.85^d^		
Height for age Z-score									
< −2SD, *n* (%)	1 (2.44)			0 (000)			> 0.9999^a^		
≥ −2SD and ≤ 2SD, *n* (%)	39 (95.12)			40 (100)			0.49^a^		
> 2SD, *n* (%)	1 (2.44)			0 (0.00)			> 0.9999^a^		
Typical symptoms									
Median days of persistent diarrhea before treatment	17			16			0.63^c^		
Median stools a day	6	4	3	6	4	2	0.43^c^	0.06^c^	** < 0.0001**^**c**^
Stools a day of ≥ 3, *n* (%)	41 (100)	36 (87.8)	34 (82.93)	40 (100)	32 (80.0)	15 (37.5)	> 0.9999^a^	0.34^b^	**0.0001**^**b**^
3–5 stools a day	18 (43.90)	25 (60.98)	30 (73.17)	18 (45)	27 (67.5)	15 (37.5)	0.92^b^	0.54^b^	**0.0016**^**b**^
6–7 stools a day	16 (39.02)	10 (24.39)	4 (9.76)	15 (37.5)	5 (12.5)	0 (0.00)	0.89^b^	0.18^b^	0.12^a^
≥ 8 stools a day	7 (17.07)	1 (2.44)	0 (0.00)	7 (17.5)	0 (0.00)	0 (0.00)	0.96^b^	> 0.9999^a^	
Presence of fecal mucus, *n* (%)	39 (95.12)	37 (90.24)	30 (73.17)	39 (97.50)	26 (65.0)	18 (45.0)	> 0.9999^a^	0.008^a^	**0.01**^**b**^
Diaper stool types 4-5B, *n* (%)	38 (92.68)	33 (80.49)	21 (51.22)	32 (80.0)	20 (50.0)	10 (25.0)	0.12^a^	0.19^b^	**0.02**^**b**^

^a^Fisher’s Exact test; ^b^Chi-Square test; ^c^ Mann–Whitney test; ^d^ *t-*test.

Significant values are in bold.

We recorded all the baseline clinical characteristics, including diarrhea duration, frequency of daily bowel movements of ≥﻿ 3 times a day, presence of fecal mucus, and diapered infant stool scale types 4–5B before treatments. No features were significantly different between the two groups (*p* = 0.63, > 0.9999, > 0.9999, and 0.12, respectively). The median stool frequency for both the Control and Clausy groups was 6 stools per day, with no significant difference observed (*p* = 0.43) (Table 1). The number of patients with watery stool types 5A & 5B was nearly identical between the two groups (*n* = 24 in the Control group, *n* = 25 in the Clausy group, *p* = 0.72). When considering the frequency of bowel movements across three categorized increasing levels (3–5 stools a day, 6–7 stools a day, and ≥ 8 stools a day), there was also no statistically significant differences observed, with *p* values of 0.92, 0.89, and 0.96, respectively.

Additionally, baseline subclinical characteristics, including the presence of white blood cells (leukocytes) and red blood cells (erythrocytes), as observed by stool microscopy, were similar between the two groups (*p* = 0.52 and 0.84). There were also no significant differences in abnormal pH values (pH ≤ 5.5) (*p* = 0.59) (Table 2). As shown in Table 2, a certain proportion of patients, nearly 30% (29.27% in the Control group, 27.5% in the Clausy group) were found to be infected with enteropathogenic microorganisms, with the number of cases per pathogen ranging from 1 to 4. There were ten microorganisms, including 7 bacterial and 3 viral species, detected in stool samples after screening the 24 most common pathogenic microorganisms causing diarrhea. Among them, the pathogenic microorganisms with the highest prevalence in both groups are *C. difficile* toxin A/B (*n* = 4 in both groups, 9.76–10.00%), followed by *Campylobacter* spp. (*n* = 3 in both groups, 7.32–7.5%), and Norovirus GII (*n* = 2, 4.88% in Control group; *n* = 3, 7.50% in the Clausy group). No difference was observed in the presence of these enteric bacteria (*p* = 0.11) and viruses (*p* = 0.26) in stool samples between the two groups. Hence, in over 60%, of cases, persistent diarrhea might be due to alternative underlying causes. In the trial, the Control group received Reverse Osmotic (RO) water, whereas the Clausy group received *B. clausii* spores (LiveSpo CLAUSY) in addition to the same standard of care treatment in the Department of Gastroenterology, Vietnam National Children's Hospital (Table 2—routine treatment and supportive treatment). The standard treatment regimen ran for 5–10 days, but it could be extended further to 15 days depending on severity of the symptoms. Patients were assessed for subclinical signs on day 0, day 3, and day 5.

**Table 2:** Subclinical characteristics of stool in persistent diarrhea children before, during, and after treatment.

Characteristic	Control group (*N* = 41)			Clausy group (*N* = 40)			*P* value		
	Before treatment	During and after treatment		Before treatment	During and after treatment		Before treatment	During and after treatment	
	Day 0	Day 3	Day 5	Day 0	Day 3	Day 5	Day 0	Day 3	Day 5
Biochemical test *n* (%)									
Erythrocyte positive (mild to severe)	10 (24.39)	2 (4.88)	1 (2.44)	9 (22.50)	4 (10.00)	2 (5.00)	0.84^b^	0.43^a^	0.62^a^
Leukocyte positive (mild to severe)	35 (85.37)	22 (53.66)	6 (14.63)	32 (80.00)	6 (15.00)	1 (2.50)	0.52^b^	**0.0005**^**b**^	0.11^a^
pH ≤ 5.5	16 (39.02)	12 (29.27)	7 (17.07)	18 (45.0)	12 (30.00)	8 (20.0)	0.59^b^	0.94^b^	0.73^b^
pH > 5.5	25 (60.98)	29 (70.73)	25 (60.98)	22 (55.0)	28 (70.0)	23 (57.5)	0.59^b^	0.94^b^	0.75^b^
24-pathogen intestinal microbial real-time RT-PCR test *n* (%)									
Bacteria	16 (39.02)			9 (22.50)			0.11^b^		
* Campylobacter* spp.	3 (7.32)			3 (7.50)			> 0.9999^a^		
*Clostridium difficile (toxin A/B)*	4 (9.76)			4 (10.0)			> 0.9999^a^		
*Enteroaggregative E. coli*	4 (9.76)			1 (2.50)			0.36^a^		
*Enteropathogenic E. coli*	2 (4.88)			0 (0.00)			0.49^a^		
*Enterotoxigenic E. coli*	1 (2.44)			0 (0.00)			> 0.9999^a^		
*Plesiomonas shigelloides*	1 (2.44)			0 (0.00)			> 0.9999^a^		
* Salmonella* spp.	1 (2.44)			1 (2.50)			> 0.9999^a^		
Virus	2 (4.88)			5 (12.50)			0.26^a^		
* Astrovirus*	0 (0.00)			1 (2.50)			0.49^a^		
* Norovirus GII*	2 (4.88)			3 (7.50)			0.68^a^		
* Rotavirus A*	0 (0.00)			1 (2.50)			0.49^a^		
Total number of infected patients	12 (29.27)			11 (27.50)			0.86^b^		
Antibiotic usage before diarrhea onset, n (%)	14 (34.15)			18 (45.00)			0.32^b^		
Treatment therapy during hospitalization									
Routine treatment	– Antibiotics: oral (e.g., Zithromax or Ciprofloxacin) or intervention (e.g., Ceftriaxone, Ciprofloxacin, Metronidazole, or Vancomycin) drugs – Oral rehydration solution (Oremut) – Zinc gluconate								
Supportive treatment	Reverse osmotic (RO) water			RO water plus *B. clausii* at 2 billion CFU/5 mL (LiveSpo CLAUSY)					
Feeding status	All children receive age-appropriate feeding ∙ For infants < 6 months: breastfeeding is preferred. If formula milk is necessary, use lactose-free milk or hydrolyzed formula if there is no improvement with lactose-free milk after 1 week ∙ For children ≥ 6 months: breastfeeding is used in conjunction with diet (lactose-free with reduced starch such as cooked cereal, vegetables, oil, and glucose. Replace milk protein with other sources like chicken, egg, or protein hydrolysate, ensuring at least 10% of calorie intake from protein) Total calories are estimated to be approximately 100 cal/kg/day								

^a^Fisher’s exact test.

^b^Chi-square test.

Significant values are in bold.

### Safety of daily supplementation with *B. clausii* spores at dosages of 8–12 billion CFU

During the observation of treatment period, we did not record any cases of adverse reactions to the LiveSpo CLAUSY product at the high dosages of 8–12 billion CFU daily, as described in the “Methods”. No cases of vomiting, unpleasant digestive symptoms (temporary increase in gas and bloating), increased thirst, or symptoms of an allergic reaction were observed in any of the participants using the product. Similar to the Control group, the body temperature of the children in Clausy group did not undergo significant changes. The median of patients’ body temperature median values remained stable at around 36.8—37.0 °C from day 1 to day 7 of measurement (Fig. S1). No participant developed complications.

### Shortening treatment time and improving treatment efficacy for diarrhea symptoms by *B. clausii* spore supplementation

**Figure 2:**
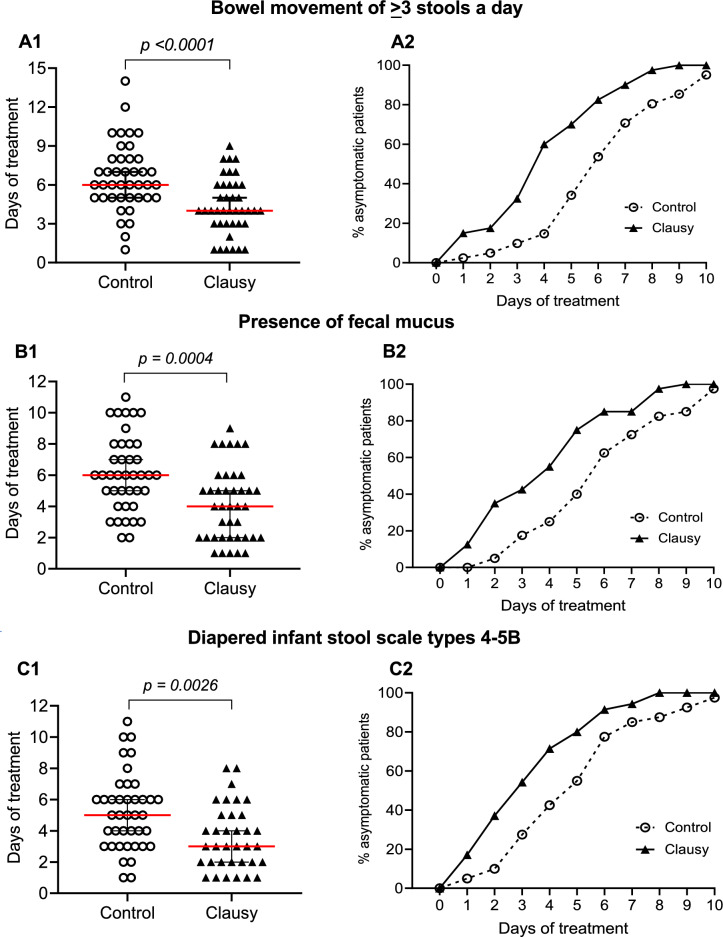
Days of treatment (**A1–C1**) for observation of three typical symptoms of diarrhea and time-dependent percentage (%) of asymptomatic patients (**A2–C2**) in the Control (dashed lines) and Clausy (solid lines) groups. Methods to test distribution was verified by Mann–Whitney test or *t*-test.

In the trial, the primary outcome is duration of treatment required to alleviate the three typical clinical symptoms of diarrhea: (i) having bowel movement of ≥ 3 stools a day, (ii) the presence of fecal mucus, and (iii) diapered infant stool scale types 4–5B. The term “days of treatment” refers to the duration (days) required to resolve a particular symptom after the first day of treatment. The results, shown in Table 1 and Fig. 2A1, demonstrate that days of treatment for the symptom of having ≥ 3 bowel movements a day occurred faster in the Clausy group than in the Control group (median: 4 days vs. 6 days). The two-day difference was statistically significant (*p* < 0.0001). After 5 days of treatment, there was a significant difference in median stools a day between the Control group (3 stools a day) and the Clausy group (2 stools a day), with a *p*-value of < 0.0001 (Table 1). Significant differences between the two groups were also observed for other two symptoms i.e., the presence of fecal mucus (2-day difference: 4 days of the Clausy group vs. 6 days of the Control group; *p* = 0.0004) and diaper stool types 4-5B (2-day difference: 3 days of the Clausy group vs. 5 days of the Control group; *p* = 0.0026) (Table 1, Fig. 2B1–C1).

As shown in Fig. 2A2, during the initial 2 days after starting treatment, there was a slight difference in the symptom of having more than bowel movements a day of children between the two treatment groups. However, from day 3 to day 10, there was a consistent and significant increasement in the percentage of asymptomatic patients without more than 3 bowel movements a day in the Clausy group. By day 3, more than 50% of children in Clausy had stopped experiencing diarrhea. The trend of improvement continued and by day 8, almost all children in the Clausy group recovered from this symptom while in the Control group, children continued to manifest diarrhea for up to 10 days. Similar data were found with the other two symptoms, including presence of fecal mucus and diaper stool types 4-5B (Fig. 2B2–C2). We calculated Days of Treatment when 50% of patients were no longer symptomatic (DT_50_) for all three symptoms compared to the Control group. Precisely, in the Clausy group, the DT_50_ for bowel movements of ≥ 3 stool a day, presence of fecal mucus, and diaper stool 4-5B-type symptoms were 3.6, 3.6, and 2.8 days, respectively. Meanwhile, DT_50_ of these three symptoms were 5.8, 5.4, and 4.6 days, respectively in the Control group (Fig. 2A2–C2). This indicates that Clausy supportive treatment were 1.6 folds, 1.5 folds, and 1.6 folds more effective than the routine treatment regarding the three major diarrhea symptoms.

**Figure 3:**
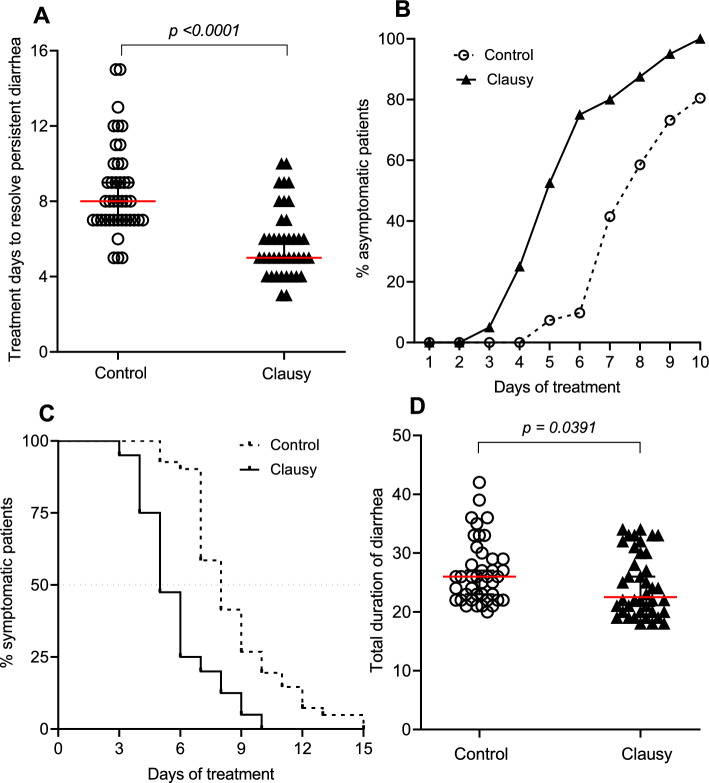
Improvement in duration and efficacy of treatment to resolve persistent diarrhea by probiotic intervention. (**A**) Overall treatment days to resolve persistent diarrhea. (**B**) time-dependent percentage (%) of asymptomatic patients (**B**) in the Control (dashed lines) and Clausy (solid lines) groups. (**C**) Kaplan–Meier analysis of estimated median time to resolve the persistent diarrhea in the Control (dashed lines) and Clausy (solid lines) groups. (**D**) Total duration of diarrhea from the onset of symptoms to the complete recovery from the disease. Methods to test distribution in Fig. 4A, D were verified by Mann–Whitney test.

Notably, as shown in Fig. 3A, the overall treatment duration to resolve persistent diarrhea in the Clausy group decreased to 5 days compared to 8 days in the Control group, and this reduction was statistically significant (*p* < 0.0001). We calculated DT_50_ when 50% patients are no longer symptomatic and the data indicate that the DT_50_ was improved by 1.6 folds in the Clausy group (DT_50_ = 4.9) compared to the Control group (DT_50_ = 7.7) (Fig. 3B). To validate these findings, we conducted Kaplan–Meier analysis to estimate the median time required for the resolution of persistent diarrhea in both groups. The curves shown in Fig. 3C illustrate the reduction in the proportion of patients with diarrhea symptoms throughout the treatment duration in the Control and Clausy groups, with a median duration of diarrhea of 8 days and 5 days, respectively. In other words, 50% of asymptomatic patients in the Control group achieved resolution on day 8, while in the Clausy group, this occurred on day 5, indicating an improvement in treatment efficiency by 1.6 folds upon probiotic intervention. These results aligned with the data presented in Fig. 3A, B. We then further examined whether the total duration of diarrhea, including the diarrhea days before treatment plus diarrhea days post treatment, of patients in Clausy group is significantly shorten than in the Control group. We found that total diarrhea duration days occurred faster in the Clausy group (median: 22.5 days) than in the Control group (median: 26 days), with a difference of 3.5 days that was statistically significant (*p* = 0.0391) (Fig. 3D). The weight gains in patients at day 3 and 5 were almost negligible in both groups during the study's observation period. No statistically significant difference in weight values was observed between the two groups at day 3 and day 5 (*p* = 0.46 at day 3; and *p* = 0.42 at day 5) (Table 1).

The above findings strongly supported our conclusion that the oral administration of high dosages of *B. clausii* spores effectively alleviated major symptoms of persistent diarrhea 2 days earlier and was 1.5–1.6-fold more effective than the standard treatment, depending on individual symptom. This helped children being free of all symptoms of persistent diarrhea 3 days sooner with a 1.6-fold increase in treatment effectiveness for complete resolution of all symptoms. It is noted that the Clausy group had 1-day longer duration of diarrhea before of treatment compared to the Control without statistically significant difference (Median: 17 days vs. 16 days, *p* = 0.63). Therefore, by shortening the overall treatment time of diarrhea by 3 days, LiveSpo CLAUSY supplemental treatment actually decreased the total duration of persistent diarrhea, from the onset of symptoms to the complete recovery, by 3.5 days.

### Evaluation of changes in blood pro- and anti-inflammatory cytokines, fecal IgA, and fecal biochemical indices by *B. clausii* spores supplementation

**Figure 4:**
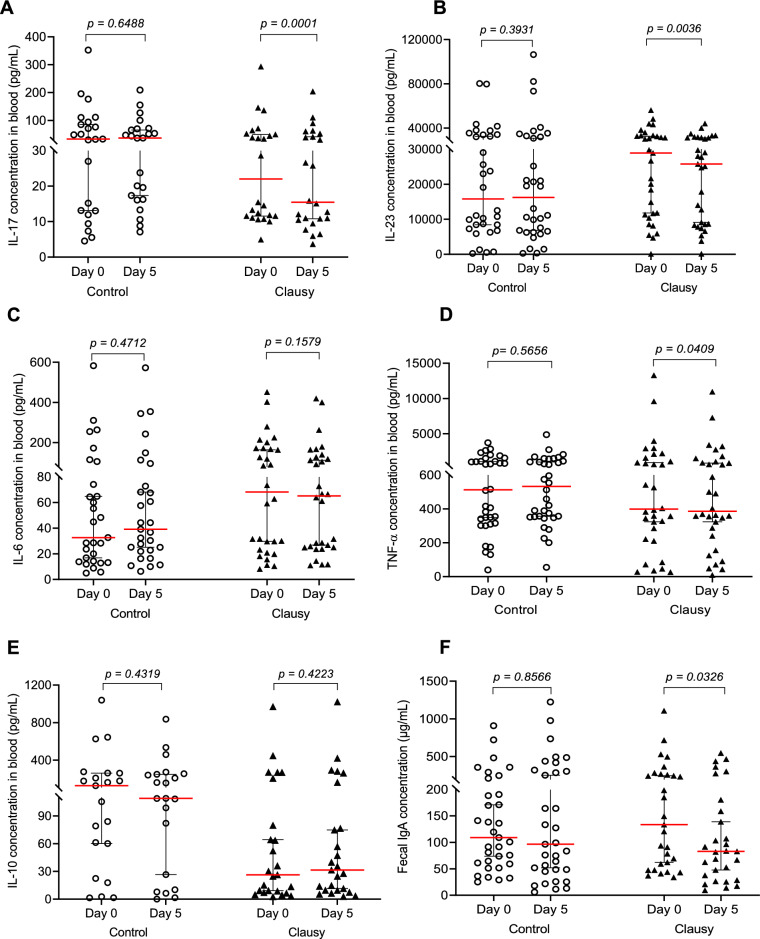
Pro- and anti-inflammatory cytokines levels (pg/mL) in blood samples and IgA levels (μg/mL) in stool samples of Control and Clausy groups at day 5 compared to day 0. The Wilcoxon test was used to calculate the median differences in IL-17 (**A**), IL-23 (**B**), IL-6 (**C**), and TNF-α (**D**) pro-inflammatory cytokine, anti-inflammatory IL-10 (**E**) cytokine, and IgA (**F**) levels at day 0 and day 5 in each group. The Mann–Whitney test was used to compared cytokine and IgA concentrations (**A–F**) between the two groups. Only samples with measurable cytokine and IgA concentrations at day 0 were included in the statistical analysis. 95% CI for median in each group and the median difference between the two groups were shown in this figure. The significance level of all analyses was set at the *p* < 0.05.

Next, we evaluated effects of the tested probiotics on the gut immune system in persistent diarrhea. We used a panel of representative cytokines to monitor levels of cytokines in peripheral blood samples together with the levels of IgA in fecal samples. Measurements of these markers serve as secondary outcomes in this study^11–19^. We selected two time points i.e., day 0 and day 5, when the clinical symptoms displayed the most apparent improvement, for analyses. The results demonstrated changes in multiple immunological markers in the Clausy group when compared to the Control group (Fig. 4). Notably, there was a significant downregulation in cytokines belonging to the Th17 pathway including IL-17 (29.76% decrease, *p* = 0.0001) and IL-23 (10.87% decrease, *p* = 0.0036), along with a statistically significant downregulation of TNF-α signalling (3.32% decrease, *p* = 0.0409). Pro-inflammatory IL-6 showed a trend of decrease (4.31%) and anti-inflammatory IL-10 showed a trend of increase (19.65%), although not statistically significant (*p* > 0.05). Treatment with probiotics showed marked decrease in IgA levels in the Clausy group (37.97%, *p* = 0.0326), while a non-significant decrease was observed in the Control (11.48%, *p* > 0.05).

The stool pH equal to or lower than 5.5 is an indicator of carbohydrate malabsorption in the colon and osmotic diarrhea^35,36^. In addition, microscopy detection of the presence of leukocytes and erythrocytes indicates intestinal inflammation and bacterial infection^37,38^. These routine hospital tests serve as subclinical indicators when considered alongside typical clinical symptoms for diagnosing diarrhea diseases. Therefore, we evaluated these features as additional secondary outcomes in this study. Among the three fecal biochemical parameters including presence of erythrocytes and leukocytes, and pH ≤ 5.5, we observed two notable trends in the Clausy group when compared to the Control group. Firstly, at day 3, there was a significant difference (*p* = 0.0005) in reduced number of cases with leukocyte positivity in the Clausy group (*n* = 6, 15.00%) compared to the Control group (*n* = 22, 53.66%). Secondly, this trend persisted at day 5, with fewer instances of leukocyte positivity in the Clausy group (*n* = 1, 2.50%) compared to the Control group (*n* = 6, 14.63%), and this difference had a *p*-value of 0.11. There were no significant differences in reduction observed for the remaining two indices at both day 3 and day 5 (*p* > 0.1) (Table 2).

## Discussion

Probiotics have been widely used throughout history and are generally regarded as safe^39^. *B. clausii* spore suspension has been well-studied for supportive treatment of acute diarrhea at the conventional dosage of two ampoules of two billion CFU/ampoule daily^27,31^. This is the first double-blind randomized controlled trial to investigate the safety and efficacy of *B. clausii* ANA39 spore probiotics at about two to threefold higher dosage of 4–6 ampoules of two billion CFU/ampoule daily (equivalent to 8–12 billion CFU daily) in treating persistent diarrhea in children aged 3 to 24 months. These high dosages were selected based on the World Gastroenterology Organization’s (WGO) recommended probiotic dosage of approximately ten billion CFU daily for the purpose of supportive treatment^40^. The results of the study showed that *B. clausii* treatment at these high dosages was well-tolerated and associated with no recorded adverse events. It is consistent with previous randomized controlled studies, which have also shown the excellent safety profile and efficacy of *B. clausii*^28,41^.

Regarding the probiotic efficacy, our study results showed that treatment with the probiotics significantly reduced in both symptomatic and overall treatment time for children with persistent diarrhea, and these effects were evident as early as day 3. The duration may be attributed to the required time for *B. clausii* spores to colonize on the gut, germinate into vegetative cells, and multiply to grow in order to balance the gut microflora. The group receiving *B. clausii* demonstrated a more notable reduction in the number of cases having the three typical symptoms associated with persistent diarrhea at two observed time points, specifically day 3 and day 5, in comparison to the control group (Table 1). The high dosage of LiveSpo CLAUSY has demonstrated its ability to reduce the duration of treatment for the three typical symptoms of diarrhea, namely bowel movement of  ≥ 3 stools a day, the presence of fecal mucus, and diaper stool types of 4–5B, by 2 days, resulting in an improved efficacy of 1.5–1.6 folds. This not only benefits the children's health but also alleviates parental anxiety and reduces the time spent on caregiving, while also lowering the cost of therapy and hospitalization. The significant reduction of 3 days in treatment time to resolve diarrhea and the overall improvement of 1.6 folds in treatment efficacy achieved through supplementation with *B. clausii* spores at such high dosages is noteworthy. This is particularly significant considering that children under three years old in low-income countries typically experience an average of three episodes of diarrhea each year^42^, and the increasing number of diarrhea cases resulting from COVID-19 infections^4^. In this study, we noted that a range of 34.15–45.00% of total enrolled patients in both the Control and Clausy groups had used antibiotics before the onset of diarrhea (Table 1). This suggests that antibiotic use may lead to AAD, particularly in cases with negative enteric pathogens. While we have not yet performed a microbiome analysis of pediatric stool samples, the reduction in duration and increased effectiveness of treatment in suspected cases of AAD suggest the potential for LiveSpo Clausy to restore gut microbiota balance in patients with dysbiosis following antibiotic use. A similar clinical study utilizing *Lactobacillus rhamnosus* GG probiotics was conducted by Basu et al., 2007^43^. Their findings revealed that the administration of additional probiotics led to a significant reduction in hospital stay by 8 days (7.3 ± 1.6 days in the *L. rhamnosus* GG receiving group vs. 15.5 ± 1.5 days in the control group; *p* < 0.05). However, the number of treatment days for the *L. rhamnosus* GG receiving group was still 1.3 days longer compared to the Clausy group. Another meta-analysis of probiotic studies involving *Lactobacillus rhamnosus* GG also showed a reduction in the duration of acute diarrhea by only one day compared to a placebo group^44^. Our data also indicated a faster improvement in supportive treatment of the number of bowel movement a day when using *Bacillus* *clausii* spore probiotics at higher dosage of 8–12 billion CFU daily, compared to previous results showing a shortened treatment time using the same *B. clausii* probiotics but at lower dosage of 4 billion CFU daily for acute diarrhea^30,45^. This difference however can be influenced by the distinct properties of clinical symptoms in patients with acute diarrhea^2,46^ and persistent diarrhea in this study. Previous studies on pediatric acute diarrhea suggest that probiotic agents are more efficient against viral (rotaviral) diarrhea, rather than bacterial diarrhea^47,48^. Therefore, a heterogeneity in the viral vs bacterial etiologies among intervention and control groups could be a confounding factor in these types of trials. In this study, the absolute numbers of total cases positive on viral detection by real-time RT-PCR appeared different between the two groups (2 cases of one-viral positivity in Control group vs. 4 cases in Clausy group including 3 cases of one-viral positivity and 1 case of two-viral positivity); however due to their relatively low percentages, the overall difference between the two groups did not reach significant levels (*p* = 0.68 for rotavirus, and 0.26 for all viruses). To further address this issue, alleviation effects by LiveSpo CLAUSY were evaluated in a pair-wise comparison of patient groups i.e., viral-positive (4 positive cases) vs viral-negative (36 negative cases) groups, or viral-positive vs baterial-positve (8 positive cases) groups. The results showed no significant differences in primary outcomes of treatment days for the three typical symptoms associated with viral infection (Table S1), suggesting that pathogens are most likely not the factor determining the effectiveness of LiveSpo CLAUSY treatment in our study. Further investigations with larger samples are required to conclusively establish whether differences exist between viral and bacterial infections in the effects of probiotic agents in the context of persistent diarrhea in children.

The intestinal mucus layer is vital for maintaining intestinal health with a dual function: (i) providing protection for the mucosa against certain microorganisms while simultaneously acting as a substrate for bacterial growth, and (ii) supplying a binding site, nutrient source, and structure. The presence of mucus in the digestive tract is an important defence mechanism against bacteria^49^. Although trace amounts of mucus in stool are normal, an excessive amount of mucus or red blood may indicate a digestive disorder. Our study showed a reduction in the mucus content of stools in both the Clausy and the Control groups during treatment time (Fig. 3B2), however the Clausy group demonstrates significant quicker reduction with shorter days of (4 days vs. 6 days) and DT_50_ value (3.6 days vs. 5.4 days) (Fig. 3B1–B2). This data was consistent with the greater decrease in IgA discharged in stool of the Clausy group (37.97%) compared to the control group (11.48%), as well as the lower proportion of cases showing leukocyte positivity in stool of the Clausy group (2.50%) compared to the Control group (14.63%) observed at day 5. The intestinal lumen contains secretory IgA (sIgA), which plays a protective role in safeguarding the intestinal epithelium against enteric pathogens and their toxins^50^. Although we cannot directly measure the concentration of IgA in the children's gut, the observed reduction in fecal mucus and IgA levels suggest that the mucus layer and IgA level on the intestinal epithelium of the patients using LiveSpo CLAUSY is effectively recovering and increase in parallel.

Although we were not able to conduct a microbiome analysis, we could expect a gut dysbiosis in all if not most of these cases of persistent diarrhea, and that the microbiota balance in these patients could be facilitated by the use of *B. clausii* spores. In accordance with this notion, there was a significant depression of the Th17 pathway evidenced by down-regulation of the cytokines IL-17 and IL-23 in the Clausy group at day 5 post-treatment, in contrast to the control group which revealed trends of increase of these cytokines. In the last decade, the gut microbiota has emerged as a potent immunoregulator with effects that go beyond the digestive system; and central to this systemic immunoregulation is the Treg/Th17 axis^16,51,52^. In particular, as the secretion of IgA from intestinal mucous is known to be promoted by IL-17^53^, the depression of the Th17 observed in patients receiving Clausy probiotics treatment might at least partially contribute into a significantly downregulated IgA level detected in their faeces. To our knowledge immunomodulatory effects by probiotics in persistent diarrhea have not been studied before. Nevertheless, our data is in line with a decrease of IL-17 reported in a recent clinical trial using the strain *B.clausii* UBBC-07 in inflammatory bowel disease^54^. These authors demonstrated a significant decrease of pro-inflammatory TNF-α and IL-6 and elevation of anti-inflammatory IL-10 post-treatment. In our study, these markers showed trends of changes but were marginally significant, except for TNF-α. Due to differences in clinical settings and probiotic products tested, we did not however expect all immunological markers in our study to show the same changes in comparison with previous clinical studies using *B. clausii* or other probiotics^54–56^.

Our study's limitation lies in the lack of statistically significant differences in both cytokine and IgA levels between the two groups at day 5 (*p* > 0.05). This may be due to patient dropouts or small sample size designed suitably for primary outcomes which are clinical symptoms, but not for secondary outcomes which are subclinical index. In addition, analysis of gut microbiota in patients was not available. The information could have helped us better understand the role of probiotics in improving the microbiota and subsequently modulating the gut immune system to support the symptomatic treatment of persistent diarrhea. Due to budget and personnel limitations, as well as time constraints in our research monitoring of a clinical trial in young children, only high dosage of approximately 10 billion CFU daily as recommended by WGO for supportive treatment was selected^40^. Therefore, future work will be performed to assess the dose-dependent effects of LiveSpo CLAUSY across multiple different test groups to determine the optimal dosage for supporting the treatment of persistent diarrhea. In a future study, we will design trial with a larger sample size with multiple dose-dependent experimental groups and perform 16S rRNA metagenome analysis using NGS (Next Generation Sequencing) to accurately assess effects and mechanisms of *B. clausii* spore probiotics at their optimal dosage.

In conclusion, supportive treatment with *B. clausii* spores at high dosages was well-tolerated with no adverse events. The Clausy group showed a significant reduction in treatment time of the three typical diarrhea symptoms by 2 days with 1.5–1.6-fold more effective compared to the Control group, resulting in 3-day shorten overall treatment time with 1.6-fold improved treatment efficacy. The analysis revealed a statistically significant 3.5-day reduction in the total duration of persistent diarrhea in the Clausy group compared to the Control group. Moreover, the Clausy group demonstrated a significant decrease in pro-inflammatory cytokines TNF-α, IL-17, and IL-23 in blood by 3.24% (*p* = 0.0409), 29.76% (*p* = 0.0001), and 10.87% (*p* = 0.0036). Simultaneously, there was an increase in anti-inflammatory cytokine IL-10 levels in blood by 19.65% and significant decrease in IgA discharged in stool by 37.97% (*p* = 0.0326) after 5 days of treatment, supporting the probiotic's potential immunomodulatory effects. This probiotic therapy may provide significant social and economic benefits in the treatment of diarrhea, especially in low- and middle-income countries where persistent diarrhea remains a severe disease.

## Methods

### Materials

The food supplement LiveSpo CLAUSY, manufactured by LiveSpo Pharma in Hanoi, Vietnam, is a reverse osmotic (RO) water suspension containing *B. clausii* ANA39 spores with a concentration of ≥ 2 × 10^9^ CFU/ 5 mL ampoule. The product was manufactured under GMP standards approved by the Ministry of Health, Vietnam (Certificate No. 18/2021/ATTP/CNG-GMP and HACCP (Certificate No. VICB 7831.6-A). Prior to the manufacture and clinical study, the strain underwent microbial and biochemical characterization, antibiotic susceptibility testing, 16S rRNA sequencing, and whole genome analysis. The summarized data include (i) microbial and biochemical characterization (Table S2); (ii) antibiotics susceptibility (Table S3); (iii) 16S rRNA sequencing analysis (Fig. S2 and S3); and (iv) sequence analysis of antibiotic resistance and toxic genes in the whole genomes of *B. clausii* ANA39 (Tables S4 and S5). The results derived from these analyses provide evidence that *B. clausii* ANA39 demonstrates safety in in-vitro test. The results also indicated that the strain is highly efficient in spore formulation (> 90%), heat-stable (> 65 °C), and can survive in both aerobic and anaerobic conditions. These favourable properties make it easy to produce the liquid-form spores at low cost, maintain quality during storage at room temperature, and remain viable to produce their effects in the gastrointestinal tract. Acute and sub-acute toxicity studies were conducted on mice and rabbit using respective dosages 250-fold and threefold higher than conventional human dosages. These studies were carried out by the Vietnam National Drug Quality Control Institute on LiveSpo CLAUSY, and they have confirmed that the product is non-toxic. The taste, smell, colour, and turbidity of LiveSpo CLAUSY and the control product (RO) are indistinguishable to nurses and patient's parents due to opaque plastic ampoule. The control and intervention products were assigned code A and C, respectively, and this information was kept confidential to most investigators and nurses (except the PI and the data analyst) and to all parents of the children.

### Ethical issues

This study was approved by the Ethics Committee in Medical Research of the Vietnam National Children's Hospital under Decision 1077/BVNTU-HDDD on 1st June, 2022 and was conducted in compliance with the ethical principles outlined in the Helsinki statement, the ICH GCP guidelines, and Vietnam Ministry of Health's regulations and standards on human subject research. All parents of pediatric patients who participated in the study were fully informed about the study and provided their consent by signing the informed consent form. Participants were free to withdraw from the study at any time upon request. The study was registered with ClinicalTrials.gov, Identifier No: NCT05812820, on 14/04/2023.

### Study design and patient collection

This was a double-blind, randomized, controlled trial, with the Control group used RO water and an experimental group (named the “Clausy” group) used the probiotics LiveSpo CLAUSY. The study lasted for 12 months, from July 2022 to July 2023, and included pediatric patients (both male and female sex) having persistent diarrhea at Department of Gastroenterology, Vietnam National Children’s Hospital. Sample size was calculated based on a hypothesis is that LiveSpo CLAUSY alleviates persistent diarrhea symptoms about 30% more effectively, as indicated by 90% of patients in the Clausy group are symptom free at day 5–9 of intervention, compared to 60% of patients in the Control group. Estimated required sample size for each group was 42 at the end of intervention (α = 0.05; power level = 0.9). Due to the high average rate (about 40–50%) of children hospitalized with persistent diarrhea among all gastrointestinal diseases in children hospitalized at the Department of Gastroenterology, Vietnam National Children's Hospital, an about 200–250 patients were estimated to participate in the screening for eligibility and 100 eligible participants (*n* = 50 per group) were randomly assigned by lottery to Control and Clausy group to reduce the risk of about 20% patient’s drop out during follow-up treatment. Eligible participants randomly assigned a study ID number from 1 to 100 to the Control and the Clausy groups. The permuted block randomization technique was applied, in which patients were allocated to blocks of random size, with a size of 2. For each block, patients were randomly allocated to Clausy or Control groups. The randomization code was computer-generated by an information technology (IT) specialist at the Pharmaceutical Department of Vietnam National Children’s Hospital, using Excel RAND function (Microsoft, WA, US), and the pharmacist dispensing the study products had access to the key. The letter A and C were assigned to the Control and the Clausy groups, respectively, and this information was also blind to all parents of children, nurses, and investigators, except the PI and the data analyst. Since no complication develop in any cases, the unblinding of randomization codes was performed only after completing the statistical analysis. The flow charge of study was shown in Fig. 1.

Inclusion criteria were:

1. Patients aged 3 to 24 months old.
2. Patients with loose stools or abnormal water on ≥ 3 times/day for ≥ 14 days and < 30 days.
3. The child's parents or caregiver understands the content of the interview questions, agrees to participate, and complies with the study protocol.

Exclusion criteria included:

1. Patients had any systemic illness other than diarrhea on admission.
2. Patients have any systemic complications during treatment.

### Questionnaires, treatment procedures, and clinical observation

The parents of the patients were asked to provide the following information about their children: full name, gender, age, obstetric history, vaccination history, history of antibiotic use, duration of diarrhea, and underlying diseases.

All participants were admitted for inpatient treatment in the wards of the Gastroenterology Department at the Vietnam National Children’s Hospital. Nurses were trained to administer a dosage of 2 ampoules (either placebo or probiotics) per administration, three times a day (totally 6 ampoules per day—“loading dose”) for initial 3 days and two times a day (totally 4 ampoules per day—“maintenance dose”) for subsequent days of treatment. The treatment duration varied depending on the severity of the illness and the patient's response to the treatment protocol. It was calculated as the day the patient recovers from all symptoms, typically ranging from 3 to 10 days. LiveSpo CLAUSY was prescribed for patients until their discharge from hospitalization, typically extending treatment time by an additional 2 days to ensure complete recovery and prevent any recurrence of the disease before hospital discharge. With the exception of patients who withdrew from the study, all patients were discharged in the afternoon of day 5 as earliest for the purpose of follow-up monitoring symptomatic clinical and subclinical indicators, even if all symptoms had resolved earlier.

Standard hospital treatment drugs were implemented based on the child's age and clinical presentation. These included (1) reversing dehydration using oral rehydration solution (ORS), (2) zinc supplementation, and (3) targeted treatment for bacterial infections using antibiotics, as briefly presented in Table 2. No anti-motility drug was prescribed for patients included in the study. For more details: (1) ORS was administered according to the World Health Organization (WHO) recommendations. For patients with diarrhea but no evidence of dehydration, ORS is used to maintain hydration by replacement of stool losses. If the stool output is minimal, ORS may not be necessary. The volume of ORS usage depends on the child's age and the patient's level of dehydration. For children under 2 years old but with no sign of dehydration, 50–100 mL should be used after each episode of diarrhea. For children with some dehydration, the dosage is 75 mL/kg body weight every 4 h, and it requires supervision and frequent reassessment. As there were no participants in a severe state of dehydration, intravenous fluid therapy was not administered. (2) Zinc supplementation was also administered according to the WHO recommendations, with a dose of 10 mg/day for 10–14 days for children bellow 6 months of age and 20 mg/day for children older than 6 months. (3) Antibiotic was administered in cases where intestinal bacterial infection was suspected. Antibiotic susceptibility tests and appropriate antibiotic treatment were provided to all patients who tested positive for enteric bacterial pathogens. This was conducted in accordance with the hospital's protocols and recommendations from the Vietnamese Ministry of Health, following the guidelines of the Infectious Diseases Society of America (IDSA).

Feeding status was implemented based on the guidelines of the hospital's Nutrition Department, following the recommendations of the Vietnamese Ministry of Health. In brief, nutritional care was provided to ensure proper nutrition for the patients. Infants under 6 months were advised to be breastfed. In cases where there was insufficient breast milk, infants were fed with lactose-free commercial formula or hydrolyzed formula if there is no improvement with lactose-free milk after one week. For children aged 6 months and older: breastfeeding should be continued and optimal feeding of mixed diet is important (lactose-free, reduced starch diet such as cooked cereal, vegetables, oil, and glucose; milk protein is replaced by chicken, egg, or protein hydrolysate, ensuring at least 10% of calorie intake from protein). Total calories are estimated to be approximately 100 kcal/kg/day.

Average weight was measured at different time points (day 0, 3, and 5), while height was measured at day 0, as it remained unchanged due to the relatively short treatment duration for diarrhea in children in this study. Weight for age Z-scores and Height for age Z-scores were assessed using the WHO child growth standards.

For primary outcomes, patients were monitored daily for typical symptoms of diarrhea, including bowel movement of > 3 stools a day, the presence of fecal mucus, status of diaper stool types 4-5B (types: 4-mucosy, stringy, more fluid than soft; 5A-watery with curds/solids; and 5B-watery without curds/solids) based on the Diapered Infant Stool Scale until their discharge. Children’s parents were instructed by doctors and nurses to perform following actions: (1) count and record their children's bowel movements within a day; (2) observe and capture photos of their child’s stools using a mobile phone to assess the presence of fecal mucus and the status of diaper stool types 4-5B based on the Diapered Infant Stool Scale atlas and reference photos of real examples of stools, which were provided and displayed at the head of the bed. In practice, the frequency of stools was counted and photos of stools was taken by children’s parents during all treatment period. The data were then provided to doctors and nurses directly in charge through discussions with the parents at every 8 a.m and 4 p.m.

Concerning adverse reactions, unpleasant digestive symptoms, increased thirst, and symptoms of an allergic reaction (if they occure) were assessed by doctors. The body temperature was directly measured by the assigned nurses, and any vomiting symptoms, if present, were monitored by the children's parents and then reported to doctors or nurses directly in charge.

### Microscopy and pH measurement of stool

These tests were conducted on day 0, 3, and 5 for all patients participating as part of the routine procedures at the Microbiology Department of Vietnam National Children’s Hospital. These procedures include (i) fresh blood smear and sedimentation rate analysis to determine presence of erythrocyte and leukocyte in stool, categorized into various levels including negativity (−), trace ( +), mild (++), moderate (+++), and severe positivity (++++); (ii) stool pH ≤ 5.5 was considered as carbohydrate malabsorption in the colon and osmotic diarrhea. These tests serve as screening criteria to ensure the selection criteria and balance of input for each group, and are secondary outcomes of this study. The tests had been standardized according to ISO 15189:2012 criteria and routinely employed in Department of Microbiology, Vietnam National Children’s Hospital.

### Multiplex real-time RT-PCR assay

Cartridge QIAstat-Dx Gastrointestinal Panel real-time RT-PCR (Qiagen, USA) with guidance from the manufacturer on a fully automated analysis system was conducted routinely on day 0 at the Department of Molecular Biology for Infectious Diseases of the Vietnam National Children's Hospital to detect 24 intestinal microbial pathogens in stool samples for the purpose of consulting on appropriate treatment therapy. The panel protocol had high sensitivity and specificity while reducing testing time, which had been standardized under ISO 15189:2012 criteria. The detected pathogens include: (1) *Entamoeba histolytica,* (2) *Cryptosporidium* spp., (3) *Giardia lamblia*, (4) *Cyclospora cayetanensis*, (5) *Vibrio vulnificus*, (6) *V. parahaemolyticus*, (7) *V. cholerae*, (8) *Campylobacter* spp. (*C. jejuni, C. upsaliensis, C. coli*), (9) *Salmonella* spp., (10) *Clostridium difficile* (*tcdA/tcdB*), (11) *Yersinia enterocolitica*, (12) *Enterotoxigenic E. coli* (*ETEC*), (13) *Enteropathogenic E. coli* (*EPEC*), (14) *Enteroaggregative E. coli* (*EAEC*), (15) *Shiga-like toxin-producing E. coli* (*STEC*), (16) *Shiga toxin-producing E. coli* (*STEC*) *serotype O157:H7*, (17) *Enteroinvasive E. coli* (*EIEC*), (18) *Shigella*, (19) *Plesiomonas shigelloides*, (20) Human Adenovirus F40/F41, (21) Norovirus GI, Norovirus GII, (22) Rotavirus A, (23) Astrovirus, and (24) Sapovirus GI/GII/GIV/GV. The procedure involved using 25–100 mg of unpreserved stool per mL of Cary-Blair transport medium, followed by transferring 200 µl of the sample into the QIAstat-Dx Gastrointestinal Panel Cartridge. Subsequently, the barcodes of the sample and the QIAstat-Dx Gastrointestinal Panel Cartridge were scanned using the QIAstat-Dx Analyzer 1.0, and the analysis was initiated. Reactions following company’s protocol used an initial denaturation at 50 °C for 20 min, 95 °C for 15 min, followed by 45 amplification and detection cycles at 95 °C for 10 s, 60 °C for 1 min, 72 °C for 30 s. After 70 min, the result was analysed by the QIAstat-Dx Analyzer 1.0. The protocol had been standardized according to ISO 15,189:2012 criteria and routinely employed in Department of Molecular Biology for Infectious Diseases, Vietnam National Children’s Hospital to detect enteric bacteria and viruses in stool samples to determine the appropriate treatment protocol for patients. These indicators are not considered as outcomes of the study.

In parallel, another real-time PCR SYBR Green assay was also conducted on stool samples on day 0, 5, and discharge day (if later than day 5) to detect the presence/absence of *B. clausii* in order to confirm the absence of *B. clausii* in stool samples at day 0 and to cross-check the proper usage of probiotics or placebo in the experimental and control groups, respectively^48^. Specific primers used for amplification of *B. clausii* were Clausii-F: 5’- AATTTTTACCGCCCTCAAG-3’ and Clausii-R: 5’- ACTTTTGGAACATGCCGAAC-3’ and real-time PCR condition was as follows: 95 °C for 10 min, amplification for 40 cycles at 95 °C for 15 s, 60 °C for 20 s, 72 °C for 30 s. The read-out standardization for *B. clausii* analysis was set at C_t_ < 35 to confirm true positive. The protocol had been developed following the ISO 17025:2017 guideline and applied for research purpose only at Department of Molecular Biology for Infectious Diseases, Vietnam National Children’s Hospital.

### ELISA for cytokine and IgA levels

As secondary outcomes of this study, the enzyme-linked immunosorbent assays (ELISA) were conducted on stool and blood samples on day 0 and 5 at the Department of Molecular Biology for Infectious Diseases of the Vietnam National Children's Hospital to determine pro-inflammatory cytokine levels (IL-6; TNF-α, IL-17, and IL-23) and anti-inflammatory cytokine IL-10 levels in blood samples, as well as IgA levels in stool samples, for evaluating changes in immune-related indicators during the treatment. The levels of the pro/anti-inflammatory cytokines and IgA were quantified by using ELISA kits following the manufacturer's instructions (R&D Systems, MN, US). The samples were measured using the SpectraMax Plus384 Microplate Reader system. The results were analysed using the SoftMax Pro 6.3 software (Molecular Devices, CA, US).

### Statistical analysis

The tabular analysis was performed on dichotomous variables using the Chi-Square test or *t*-test. In cases when the expected value of any cell is below five, Fisher's exact test was used. Continuous or independent variables were compared using either the Wilcoxon test or the Mann–Whitney test when data were not normally distributed. Statistical and graphical analyses were performed on GraphPad Prism v8.4.3 software (GraphPad Software, CA, USA).

### Supplementary Information

Supplementary Information.

## Data Availability

The datasets used and analysed during the current study available from the corresponding author on reasonable request.
