# Supplementary material for: Promising clinical and immunological efficacy of Bacillus clausii spore probiotics for supportive treatment of persistent diarrhea in children
Source: Sci Rep. 2024 Mar 18;14:6422. doi: 10.1038/s41598-024-56627-9 (PMC10944834; doi:10.1038/s41598-024-56627-9)
Supplement: Supplementary file 1 — Supplementary Information. [file 41598_2024_56627_MOESM1_ESM.pdf]

Supplemental data

1. Supplemental tables

Table S1. Median treatment days for typical symptoms of diarrhea in viral-positive, viral-negative, and bacterial-negative subgroups of the Clausy group

**Clinical characteristic**	**Viral positive (*N = 4*)**	**Viral negative (*N = 36*)**	**Bacterial positive (*N = 8*)**	***p value***	
	(1)	(2)	(3)	(1) vs. (2)	(1) vs. (3)
Median treatment days for stools a day of ≥3	4.5	4.0	4.0	0.5341[a]	0.5406[a]
Median treatment days for presence of fecal mucus	2.0	4.0	4.5	0.1546[b]	0.2323 [a]
Median treatment days for diaper stool types 4-5B	3.0	3.0	3.0	0.6549[b]	0.5080 [a]
Median treatment days to resolve persistent diarrhea	5.0	5.0	5.5	0.476[b]	0.5798[b]

Notes: [a]*t*-test

[b]Mann-Whitney test

Table S2. Microbial and biochemical characterization of *B. clausii* ANA39

Characteristics	Value
Sporulation efficiency (%)	90
Heat stability of spores (°C)	65
Width size of vegetative cell (μm)	< 1 μm
Amylase	++++
Caseinase	+++
Lipase	++
Catalase	+
Gelatinase	+
Optimal temperature (°C)	37
Optimal pH	8.0
6.5% NaCl, 50°C	-
Aerobic/Anaerobic	+++
Anerobic	+++
Hemolysis	γ (No)
VP Test	-
Closest match*	*B. clausii* (99.5%)

-, negative; +, weak or positive; ++, average; +++, good/high; ++++, very good/very high.

**Using 16S rDNA sequence analysis in this work. The similarity score is shown in brackets.*

Table S3. Antibiotic susceptibility of *B. clausii* ANA39

**Antibiotic discs (µg)**[*]	***B. clausii* ANA39**[+]
Ampicillin (10)	27.04 ± 1.3 (S)
Chloramphenicol (30)	18.47 ± 0.20 (S)
Ciprofloxacin (5)	30.89 ± 0.61 (S)
Clindamycin (2)	0 (R)
Cotrimoxazol (25)	34.50 ± 0.95 (S)
Erythromycin (15)	0 (R)
Gentamicin (10)	28.22 ± 0.39 (S)
Kanamycin (30)	24.84 ± 0.04 (S)
Neomycin (30)	24.91 ± 0.13 (S)
Rifampicin (30)	39.06 ± 0.68 (S)
Streptomycin (10)	6.51 ± 0.46 (R)
Tetracycline (30)	27.99 ± 0.14 (S)
Trimethoprim (5)	39.83 ± 0.72 (S)
Vancomycin (30)	22.41 0.22 (S)

[*]*Antibiotic-impregnated discs (6 mm) with amount in µg shown in brackets.*

[+]*Diameter of inhibition zones from three individual experiments. S, sensitive; I, intermediate resistant; R, resistant.*

Table S4. Sequence analysis of antibiotic resistance genes in *B. clausii* ANA39 genome

Resistance gene	% Identity	Query / Template length	Contig	Position in contig	Predicted phenotype	Accession number
ant(4')- Ib	98.83	771 / 771	000000F |arrow	81510..82280	Aminoglycoside resistance	AJ506108
erm(34)	96.04	833 / 846	000000F |arrow	1033445..1034277	Macrolide resistance	AY234334
cat	96.79	685 / 687	000000F |arrow	2589949..2590625	Phenicol resistance	AY238971

Notes: The three genes including *ant(4')-Ib* classified in Aminoglycoside antibiotic group, *erm(34*) classified in Macrolide antibiotic group, and cat classified in Phenicol antibiotic group were found. They may belong to acquired antibiotic resistant genes. The presence of the two genes *ant(4')-Ib* and *erm(34*) are consistent with the streptomycin and erythromycin resistant phenotype of *B. clausii* ANA39 indicated by the diffusion dics assay. Although *cat* is available in the genome, its expression level may be low so that the strain is still sensitive to chloramphenicol. Interestingly, clindamycin resistance gene was not found in the genome of *B. clausii* ANA39, suggesting that the strain is intrinsic resistance to clindamycin.

Table S5. Sequence analysis of toxin genes in *B. clausii* ANA39 genome

No	Gene	Specific sequence amplified by PCR	Number of gene detected by whole genome sequencing
1	Hemolysin B (*hbl*B)	Not Detectable	0
2	Non-hemolytic enterotoxin A (*nhe*A)	Not Detectable	0
3	*nhe*B	Not Detectable	0
4	*nhe*C	Not Detectable	0
5	Cytotoxin K (*cyt*K)	Not Detectable	0

2. Supplemental figures

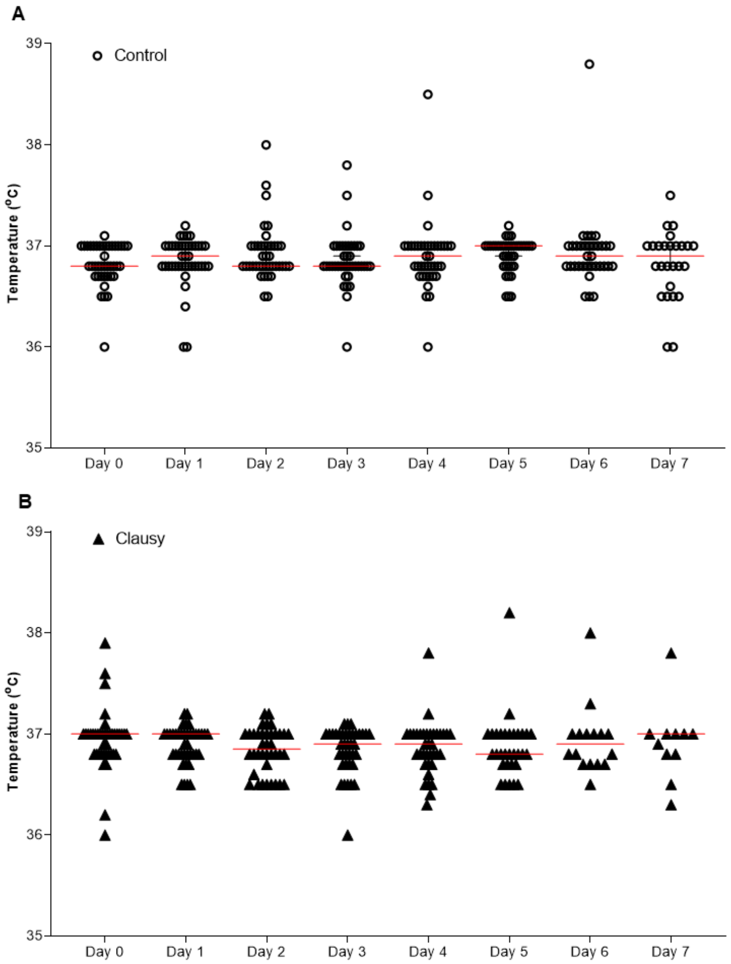

3.

Fig. S1. Average values of recorded changes in temperature between before and after oral administration with RO water (A) and LiveSpo CLAUSY at the daily dosages of 8-12 billion CFU (B), over 7 days of measurement.

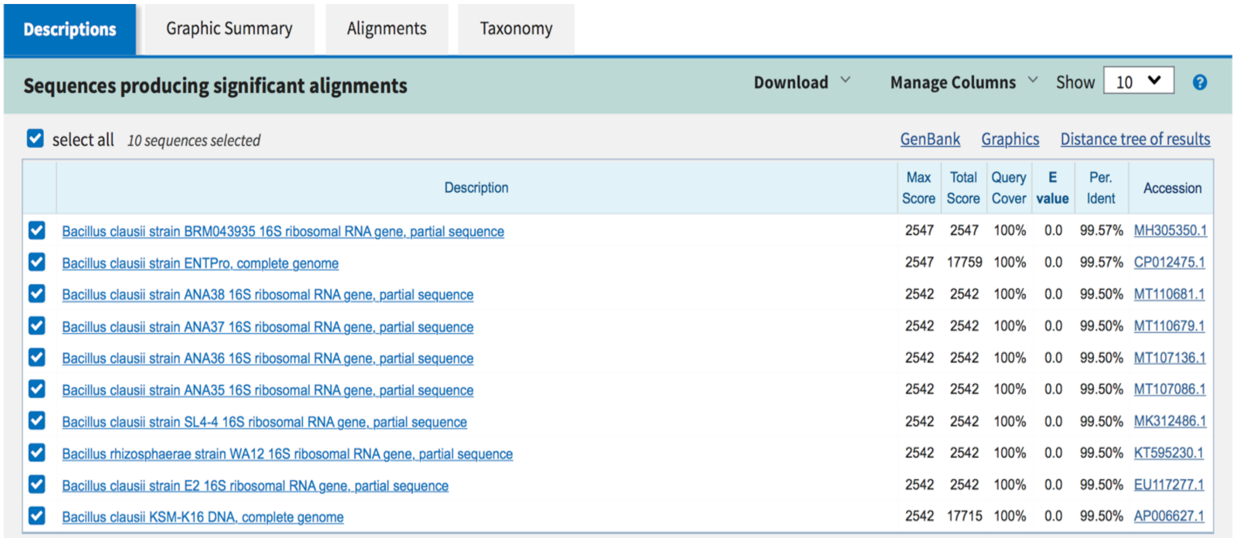

Descriptions | Graphic Summary | Alignments | Taxonomy

Sequences producing significant alignments Download Manage Columns Show 10

select all *10 sequences selected* GenBank Graphics Distance tree of results

Description	Max Score	Total Score	Query Cover	E value	Per. Ident	Accession
Bacillus clausii strain BRM043935 16S ribosomal RNA gene, partial sequence	2547	2547	100%	0.0	99.57%	MH305350.1
Bacillus clausii strain ENTPro, complete genome	2547	17759	100%	0.0	99.57%	CP012475.1
Bacillus clausii strain ANA38 16S ribosomal RNA gene, partial sequence	2542	2542	100%	0.0	99.50%	MT110681.1
Bacillus clausii strain ANA37 16S ribosomal RNA gene, partial sequence	2542	2542	100%	0.0	99.50%	MT110679.1
Bacillus clausii strain ANA36 16S ribosomal RNA gene, partial sequence	2542	2542	100%	0.0	99.50%	MT107136.1
Bacillus clausii strain ANA35 16S ribosomal RNA gene, partial sequence	2542	2542	100%	0.0	99.50%	MT107086.1
Bacillus clausii strain SL4-4 16S ribosomal RNA gene, partial sequence	2542	2542	100%	0.0	99.50%	MK312486.1
Bacillus rhizosphaerae strain WA12 16S ribosomal RNA gene, partial sequence	2542	2542	100%	0.0	99.50%	KT595230.1
Bacillus clausii strain E2 16S ribosomal RNA gene, partial sequence	2542	2542	100%	0.0	99.50%	EU117277.1
Bacillus clausii KSM-K16 DNA, complete genome	2542	17715	100%	0.0	99.50%	AP006627.1

Fig. S2. BLAST analysis of *B. clausii* ANA39 (B).

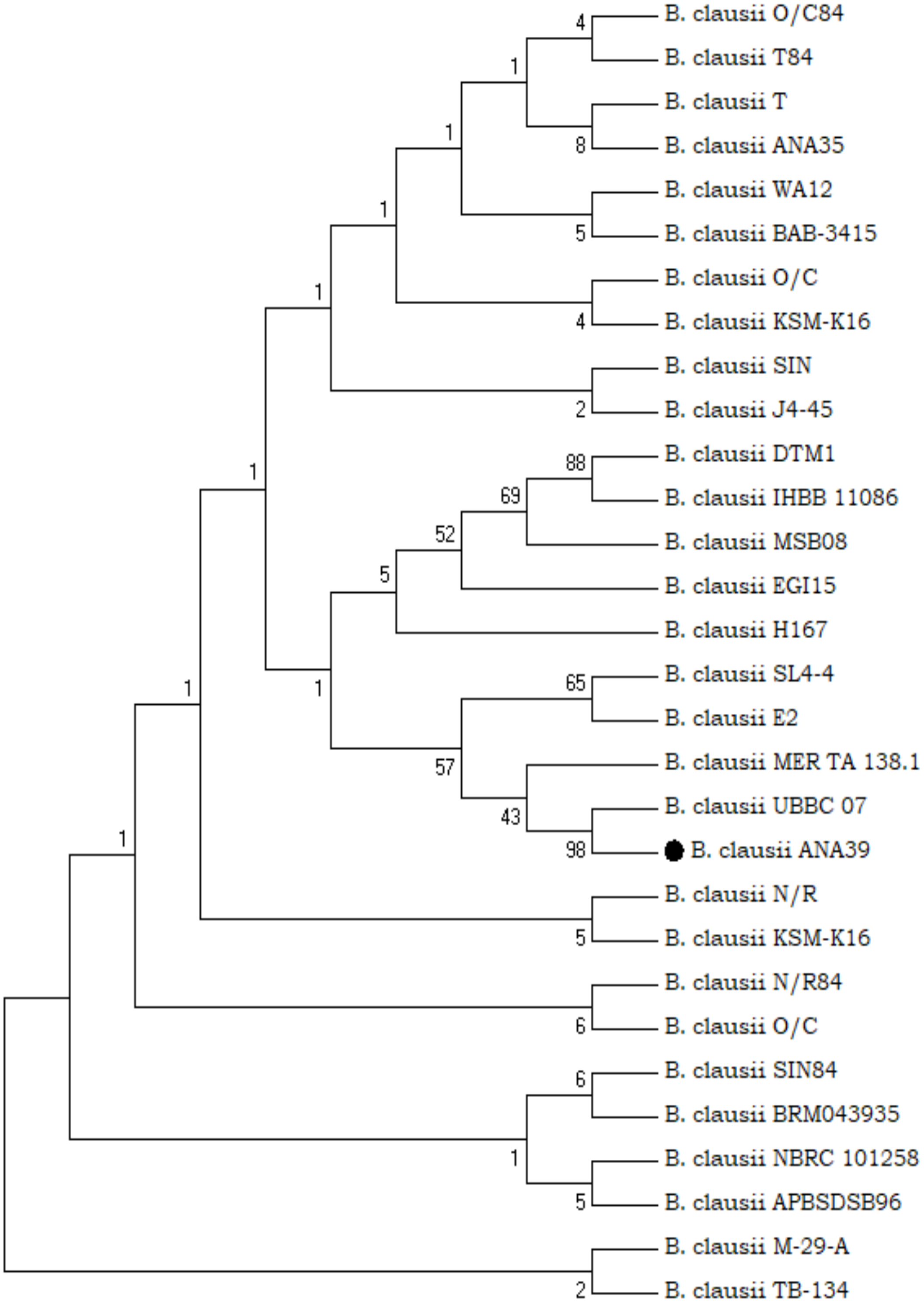

Fig. S3. Phylogenetic tree for *B. clausii* ANA39
